# End-to-End Transcript Alignment of 17th Century Manuscripts: The Case of *Moccia Code*

**DOI:** 10.3390/jimaging9010017

**Published:** 2023-01-13

**Authors:** Giuseppe De Gregorio, Giuliana Capriolo, Angelo Marcelli

**Affiliations:** 1 Department of Information and Electrical Engineering and Applied Mathematics, University of Salerno, Via Giovanni Paolo II, 132, 84084 Fisciano, Italy; 2 Department of Cultural Heritage, University of Salerno, Via Giovanni Paolo II, 132, 84084 Fisciano, Italy

**Keywords:** historical handwritten document processing, text-line segmentation, word segmentation, transcript alignment

## Abstract

The growth of digital libraries has yielded a large number of handwritten historical documents in the form of images, often accompanied by a digital transcription of the content. The ability to track the position of the words of the digital transcription in the images can be important both for the study of the document by humanities scholars and for further automatic processing. We propose a learning-free method for automatically aligning the transcription to the document image. The method receives as input the digital image of the document and the transcription of its content and aims at linking the transcription to the corresponding images within the page at the word level. The method comprises two main original contributions: a line-level segmentation algorithm capable of detecting text lines with curved baseline, and a text-to-image alignment algorithm capable of dealing with under- and over-segmentation errors at the word level. Experiments on pages from a 17th-century Italian manuscript have demonstrated that the line segmentation method allows one to segment 92% of the text line correctly. They also demonstrated that it achieves a correct alignment accuracy greater than 68%. Moreover, the performance achieved on widely used data sets compare favourably with the state of the art.

## 1. Introduction

For about twenty years, the manuscript, book, and documentary heritage have been the subject of systematic digitization campaigns aimed at guaranteeing profitable preservation and more immediate consultation, regardless of the physical places of their conservation. In addition, those campaigns ensure accurate research through the preparation of metadata sets that describe to various extents the document content and encode the codicological-paleographic characteristics, as well as educational applications, through the creation of tools dedicated to a wider audience. In this regard, we cannot fail to mention the digital libraries DigiVatLib [1], for the codes of the Vatican Apostolic Library, Gallica [2], dedicated to the manuscripts of the Bibliothéque Nationale de France, E-Codices [3], an exemplary realization of the handwritten testimonies preserved in Switzerland, Manuscripta Mediaevalia [4], containing digitized codes mainly from German libraries, the Internet culturale portal [5], for browsing through the digital collections of manuscripts held by Italian national libraries.

Simultaneously with the progress of this substantial digitization and metadating operations, the request to extrapolate the data contained in the manuscripts of these collections is becoming increasingly pressing and, therefore, it is necessary to move towards semantic management of information, providing sophisticated software for the automatic reading of the manuscript text in support of scholars and cataloguers in particular.

In recent years, machine learning techniques, and particularly deep learning ones, have been largely adopted for the automatic processing of historical documents, and have shown remarkable performance in different tasks, such as image quality enhancement, text-line segmentation, keyword spotting and character recognition [6], as well as in handwriting text recognition [7]. Deep learning techniques, however, leverage on large annotated training sets, extracted from the collections to transcribe, which requires huge human efforts to be produced. While these efforts can be justified in the case of large collections, i.e., collections including hundreds of pages, they are impractical in the case of small ones, i.e., collections including a few tens of pages, since the number of pages that must be manually annotated for building the training set may be a significant part of the whole collection, thus drastically reducing the advantages of having the remaining part of the collection automatically processed for the intended purposes. Moreover, the annotation of the training set may involve, and very often it does, skilled scholars, and, at the same time, the setup and execution of the training procedure require computational resources and advanced technical skills that may not be available at small libraries, local museums, churches archives and other cultural institutions.

The efforts in the field of transcription of ancient manuscript documents are reflected in the increasing availability of digital versions of texts. For this reason, images of handwritten documents together with their digital transcripts are today easily accessible to a wide audience. However, the digital transcript is not always linked to the manuscript image, making it difficult to locate the parts of the image that correspond to a particular part of the transcript. Thus, tools for transcript alignment, i.e., the automatic linking of the digital transcription with the parts of the digital image of the manuscript where it appears, would be of great help to scholars and historians who have to work with different versions of documents and/or writing styles, as well as for engineering a semi-automatic procedure for building the training set with minimal annotation, as suggested, for instance, in [8].

Moving from these observations, we present an end-to-end solution for the transcription alignment of historical handwritten documents that has been designed and implemented by pursuing a learning-free approach. The proposed solution exploits, to a large extent, information that are automatically extracted from the document transcription, such as the number of text lines in the document, the number of words in each text line, and the number of characters of each word of the transcription. It incorporates a line-level segmentation of the page image capable of extracting text lines with a horizontal direction but a curved baseline and envisages the intertwining between transcript alignment and word-level segmentation to handle both under- and over-segmentation errors at the word level.

In the remainder of the paper, Section 2 reviews the relevant literature for transcript alignment, Section 3 describes the data collection that served as a case study for driving the design, and Section 4 presents the proposed methods by describing the text line segmentation algorithm in Section 4.2, and the alignment method in Section 4.3. Section 5 reports the results of experiments we have conducted for performance evaluation, which are discussed in Section 6. In Section 7, we summarize the main features of the method, the major outcomes of the experimental work, and outline our future research on this topic.

## 2. State of the Art

One of the first methods for image-text alignment was proposed by Tomai et al. [9]. The method works with segmented text lines at the word level and proposes an interpretation for each segmented area using a text recognition module using a limited dictionary that considers only the words present in the transcription. A dynamic programming algorithm searches for the best match between images and transcripts, achieving a percentage of 72% correct alignment.

Kornfield et al. [10] propose to align word segmentation boxes to transcription words using a Dynamic Time Wrapping algorithm. The method achieves a correct alignment of 75.40% when working with lines of text.

Rothfeder et al. [11] approach the problem as an alignment problem between two sequences by using a linear Hidden Markov Model (HMM) and applying the Viterbi algorithm to achieve an alignment that yields a correct alignment percentage of 72.80%.

Toselli et al. [12] use a similar approach based on an HMM and the Viterbi algorithm, but together with a text recognition module whose dictionary is limited to the words present in the text line transcription. They can achieve 92.80% of correct alignment in their best results. Moreover, in this case, as in [9], the performance depends on the word recognition module, and their application, therefore, depends on the possibility of applying this recogniser to the input data.

Zinger et al. [13] first perform a word-segmentation and then the segments are matched with the transcriptions. The word segmentation is performed by analyzing the longest spaces between parts of the handwriting line defining a gap metric that takes into account the length of the words. This results in a cost function that must be minimized to obtain correct alignment. The results demonstrate a 69% of correct alignments.

Indermuehle et al. [14] include a feature extraction step to use an HMM. The method achieves the best results with a training set of 2500 words correctly aligned to about 65%. Finally, a model trained on the public IAM dataset is tested. Combining the results with the previous system leads to a correct alignment of almost 95.5%. However, this result requires a training phase with a dataset of collected words to perform the alignment.

Stamatopoulos et al. [15,16], segment the document in lines of text using the Hough transform and the number of lines obtained from digital transcription. Each line is then segmented at the word level by analysing the white spaces and considering the number of words in the transcription of the text line. In [15], a manual correction phase of the alignments is performed to obtain an error-free solution. In doing so, the authors show that it is possible to save up to 90% of time compared to manually aligning 97.21% of words. In [16], the authors add an additional character-level step that brings the correct alignment to 99.48%.

Leydier et al. [17] avoid the segmentation and learning phase. The method consists of extracting the characters from the image of a whole line of text and using the Levenshtein distance to align with the transcription. The rate of correct alignment of the method is almost 73%.

Romero-Gòmez et al. [18] use a dynamic programming algorithm to align the results of a word segmentation step. An HMM text recognizer flanks the word-level segmentation, and the alignment confidence is weighted using the Levenshtein distance. The method achieves percentage correctness of alignment of about 75.5%.

Ziran et al. [19] propose to combine an object detection deep architecture, used for word location, with a dynamic programming technique to perform the alignment. The method is tested on early printed pages of the Guttenberg Bible, showing the ability to align 90% of the words.

Torras et al. [20] develop a Seq2Seq model together with an attention mechanism to align each symbol in the transcript of historical ciphered manuscripts. The architecture is trained to identify symbols, and the results show that the network can detect more than 90% of the symbols.

## 3. The Manuscript Collection

As a case study to drive the development of our method, we have used a documentary manuscript from the seventeenth century. Its writing is part of the bastard-Italic typologies widely used in the field of the chancellery in the sixteenth-seventeenth centuries, realized in brown ink, sometimes lighter, and carried out mainly by a single hand. The pages are well-readable, and their layout is rather regular. The manuscript, entitled *Code of the Moccia family. Privileges, Investitures, Announcements and Ordinances, 1449–1610*, is currently preserved in the Archives of the Salerno-Lucan province of the Friars Minor based in the convent of the Holy Trinity in Baronissi (Salerno, Italy) [21]. From now on, we will refer to this collection as the *Moccia Code*.

Being part of the typology of the book-document and, in particular, of the cartulary of which, in addition to structure and contents, also purposes and functions are taken up, especially those of “I remember for future reference”, and of documentary conservation, it formed a sort of family “archive-casket” and *munimen*, as a collection of documents certifying the rights and prerogatives of the family to be presented on the occasion of possible summons to court, or to verify the “legitimacy of the titles owned by the holders of the offices”, that could be useful, in short, for the defence in the inquisitorial and trial phase of some representative of a Neapolitan family, the Moccias, that held the office of Portolania in the Kingdom of Naples from the fifteenth century and throughout the Modern Age.

In addition to three sovereign documents issued by Alfonso il Magnanimo, Ferrante I and Federico d’Aragona relating to the conferment, the confirmation and new concession of the privilege of “mastro portolano” and procurator of the province of Terra di Lavoro to the members of the Moccia family, there are transcripts, mainly in the form of a simple copy, administrative documents (instructions for officers, orders, provisions) and judicial notices (*acta*, subpoenas, decrees, execution of sentences), produced by the main magistracy and provincial offices of the Kingdom of Naples between the 15th and the early 17th century. Probably the copy work took place directly from originals or, in any case, from loose documentation in possession of the client (or recipients) or that was temporarily available for recording. About thirty documents contain the registration data of the original document at the Royal Chamber of the Sommaria, at the Records Office of the Aragonese kings and that of the viceroys and in the office of the “mastro portolano”.

The book was probably commissioned by one of the last exponents of the family, perhaps a certain Giovanni Simone Moccia, whose titles of possession were contested and who was accused of abuses in the exercise of the Portolania Office. Here, the transcription does not provide for the completion of the abbreviated words although, occasionally, the omitted nasals and the development of special graphic signs relating to the enclitic *-ue*, to the ending *us*, *q* and *p* has been reported in round brackets.

**Figure 1:**
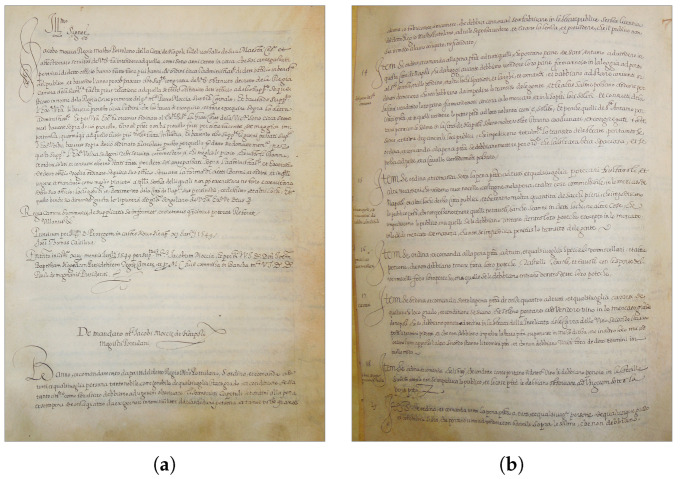

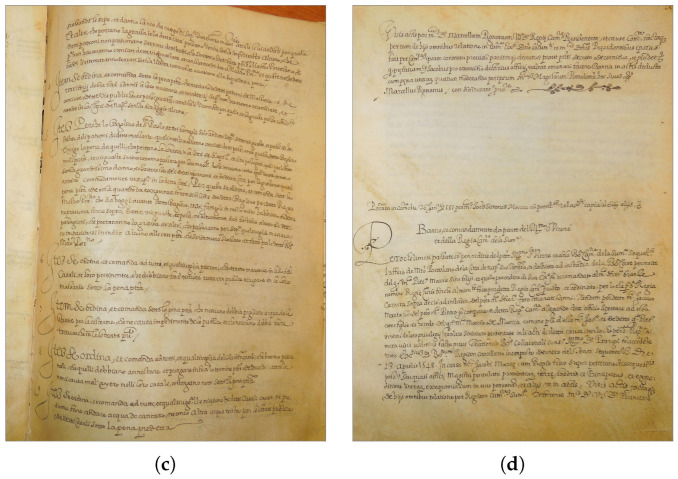
Examples of pages from the Moccia Code: (**a**) f. 6r; (**b**) f. 7v; (**c**) f. 9r; (**d**) f. 10r.

As Figure 1 shows, the Moccia Code contains mostly textual documents, thus simplifying the text line segmentation, but represents a very challenging testbed for transcription alignment, due to the irregular line spacing (Figure 1a), non-uniform lighting conditions (Figure 1b), bending text-lines (Figure 1c), non-uniform background due to ageing (Figure 1d). Moreover, there are overlapping ascenders and descenders, handwriting is small in size, the inter-word space is very irregular, and abbreviations are widely used throughout the documents.

## 4. Method

**Figure 2:**
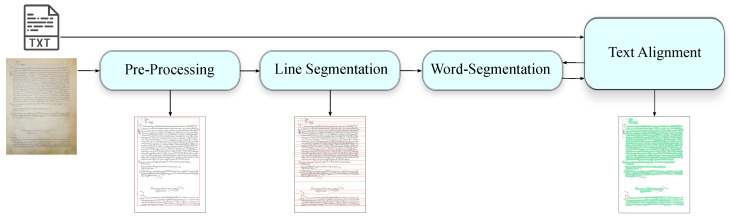
The workflow of the entire process: The input consists of the colour image of a document and its transcription. The document is pre-processed by creating its black and white version and identifying the text area. Then follows the segmentation into lines of text and finally the alignment of the transcription.

The end-to-end solution we have designed is depicted in Figure 2. The process workflow encompasses four stages, each of which articulates into several steps, as it is described below.

The input is the digital colour images of the pages that were made available by the collection hosting institution together with their line-by-line transcription, and the output is a data structure linking the transcripts to the bounding boxes of the corresponding word images in each text line. The data structure is exploited by a GUI (Graphical User Interface) that allows one to formulate a query by typing the transcript and returns the bounding box of the (hypothesized) corresponding images in the text lines.

### 4.1. Image Preprocessing

**Figure 3:**
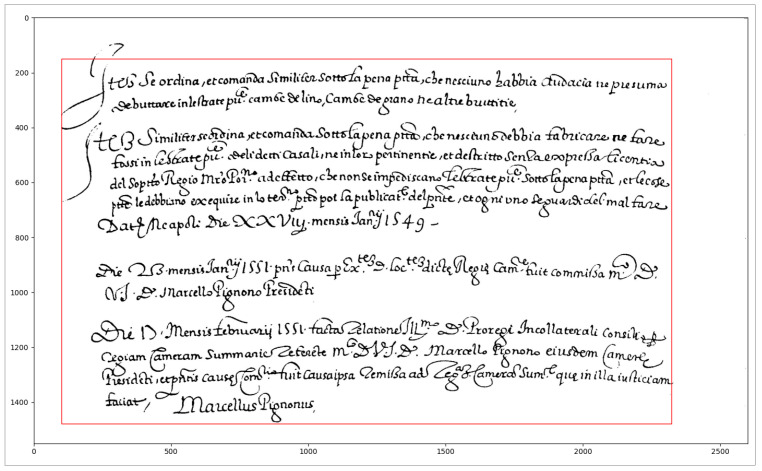
Examples text area detection: the figure shows in the red rectangle the text area detected in a document image.

The preprocessing prepares the image for the subsequent segmentation stage. First, a Gaussian filter is applied to the image to reduce noise and blur ink strokes. Then, the colour image is transformed into a binary image by using the algorithm presented in algorithm [22] with a window size of 75 × 75 pixels. Eventually, the text area is detected, by computing the horizontal projection profile (HPP) and vertical projection profile (VPP) of the foreground pixels and finding the regions of the histogram corresponding to mostly black rows/columns of the image, as shown in Figure 3. In the subsequent steps, only the pixels within the text area are considered.

### 4.2. Line-Level Segmentation

Locating the text lines within a page is a preliminary step required by most historical handwritten documents applications, such as keyword spotting, writer/script identification, document dating and handwriting recognition. It is particularly complex in historical handwritten documents because the lines may touch or overlap, be skewed or with curved baselines, the space between lines may vary along the page, and the characters may exhibit variable sizes. On the other hand, the performance of most of the approaches proposed for specific applications, as those mentioned above, would undoubtedly benefit from better segmentation. This is the reason why, although one of the earliest algorithms for document image segmentation was proposed more than 40 years ago [23], it is still an active research field, whose advancements have been presented in surveys [6,24,25,26,27], and state-of-the-art performance assessed through competitions regularly held at the International Conference on Document Analysis and Recognition [28,29,30,31]. As the most recent surveys show, in the last decade the adoption of deep learning techniques has led to great performance improvements, but they are not suitable for small collections, as discussed in the introduction.

Among the learning-free algorithms that have been proposed in the literature, the one presented in [32] separates subsequent text lines even when they partially overlap is fairly simple to implement, quite fast, and robust for different kinds of handwritten documents. However, it cannot handle text lines with curved baselines. To overcome these limitations, we have kept the idea of reformulating the text line segmentation as a path planning problem to be solved by using the A* algorithm but adopted different criteria to define the search areas for the A* algorithm by taking advantage of the information provided from the available transcriptions, as described in the sequel.

In the original method, the authors use the A* search algorithm to identify the boundaries of each text line. To identify the position of the text lines, the method computes the Horizontal Projection Profile (HPP) of the entire text area and locates the centres of the text lines in correspondence with the HPP local maxima, and thus the part of the image between two consecutive maxima of the HPP is the search space to find the cut boundary separating the two lines of text. The cut boundary is defined by applying the A* algorithm for finding the shortest path among those connecting the leftmost and the rightmost white pixels of each image row within the search area, using the squared Euclidean distance between two nodes of the path to weigh the different paths. As the goal is segmenting the page into lines of text, the black ink pixels are considered obstacles, and the path search will try to bypass them to connect the origin and destination points.

The major drawback of the method by Surinta et al. [32] is certainly its intrinsic inability to segment documents containing lines of text with a curved baseline that cannot be separated by a horizontal line. This is because the first phase of the method is based on identifying the search spaces by analysing the horizontal black pixel projection profile on the whole image. If the text lines have strongly curved baselines, it is not possible with the projection technique to satisfactorily identify the different spaces between the text lines.

**Figure 4:**
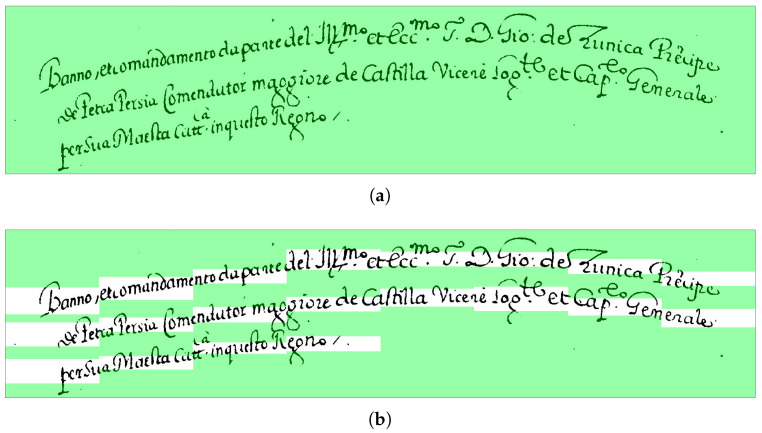
Examples of detection of search areas for text line segmentation: green areas represent the search areas detected by computing the histogram on the whole image (**a**) or on each stripe (**b**).

To overcome the issue, we conjectured that the identification of the search spaces for each part is possible by looking at the projections not of the whole image but of different successive vertical regions, called stripes. By applying the A* search on each stripe, we approximate the (curved) baseline with a step function, whose size step corresponds to the horizontal size of the stripes. Figure 4 shows an example where, calculating the HPP does not allow one to identify of any search area, and thus will not segment the text area (a), whereas the computation of the histogram on each stripe leads to as many search areas as the number of text lines in each stripe (b). In the implementation, the number of stripes *S* is fixed empirically, as will be explained in Section 5.

Below, we report the procedural steps of our text line segmentation algorithm:

1. **Divide the image into stripes.**

The image of the text area is divided into *S* stripes of the same width, in such a way that the sum of the widths of the stripes equals the width of the document image.

2. **Find the horizontal projection profile**.

For each stripe, the HPP is calculated. The profile analysis allows for identifying the text line’s position in the studied region, assuming that each peak corresponds to a text line. If the number of lines of the document is known, as in the case considered here, all the HPP local maxima can be sorted according to their values in descending order, and as many of them as the number of text lines of the document selected as the centre of the text lines. In case the histogram contains a number of peaks lower than expected, it means that not all the lines are present in the considered stripe, as in the case when the ink of the handwritten text does not extend over the entire line. In these cases, all detected peaks will be selected, even if fewer than expected. Once the peaks are identified, the space between the different peaks represents the search area separating the lines of text.

3. Carry out A* path planning along the search areas in each stripe.

**Figure 5:**
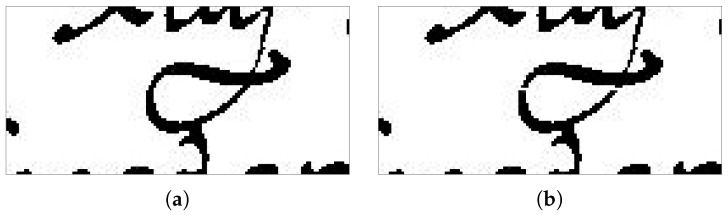
Examples of insurmountable obstacle: (**a**) An example of an insurmountable obstacle, (**b**) An example of a modified insurmountable obstacle: note the path of white pixels inserted in the middle of the ink track for allowing crossing the obstacle.

Once the different search areas have been identified for each stripe, the A* algorithm is run to find the cutting boundaries for each search area. In the original algorithm, the presence of overlapping ascenders and descenders, as illustrated in Figure 5a creates an unsurmountable obstacle to the path finding, as it is impossible to define a path without crossing them. To deal with the issue, Surinta et al. [32] modify the algorithm A* allowing obstacles to be traversed, and leave to the cost function to model whether or not an obstacle should be crossed. However, this makes the algorithm more complex and increases the difficulty of calculating the cost function.

In our implementation, we preferred to add a preliminary phase to the path planning, which consists of identifying the obstacles as the region of HPP between two local maxima, i.e., the search space for the A* algorithm, that does not contain any white row, and then tunneling through them by inserting a path of white pixels in the row of the image located in correspondence of the centre of the search area, so that the A* path planning algorithm can find a valid path. Figure 5 shows an insurmountable obstacle modified by a sequence of white pixels in the middle of the ink track.

4. **Connect the cutting boundaries between adjacent stripes.**

The final step is to combine the results of the different A* for each stripe with the results of the algorithm in the immediately adjacent one, and so on. In this way, cutting boundaries are obtained that traverse the entire text area.

**Figure 6:**
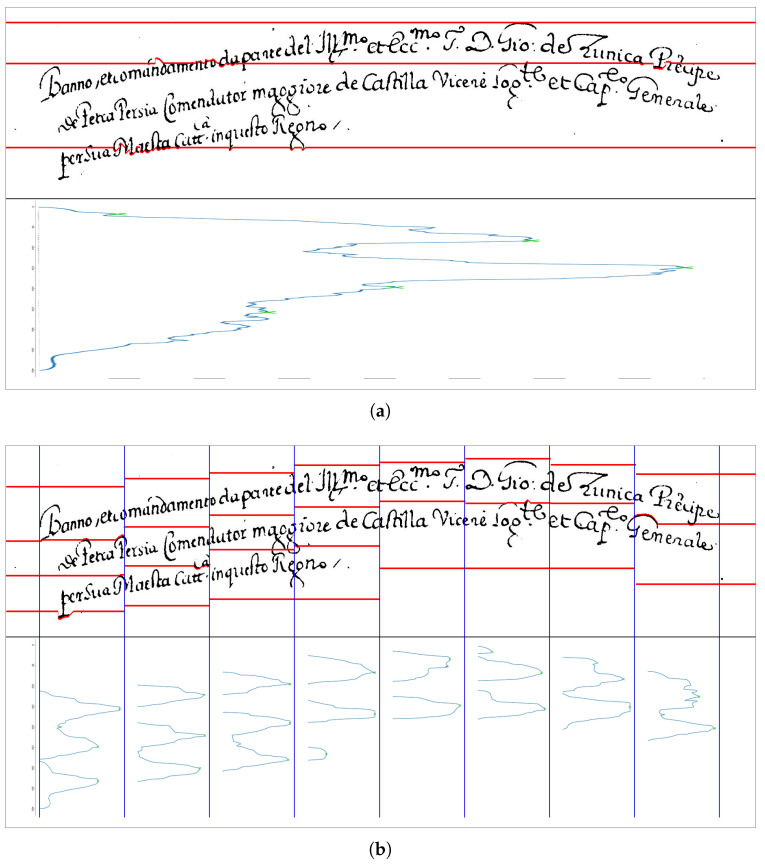
Text-line segmentation in case of lines with curved baseline: (**a**) the result of the original algorithm (S=1); (**b**) the result of our algorithm (in the case S=8).

Figure 6 shows the HPP computed on the whole text area (a), and the HPPs computed on 8 different stripes (b). In the latter case, it is much easier than in the former to identify the HPP local maxima. It is also worth noting that in the four rightmost stripes only two local maxima are detected because the handwriting of the last line does not extend over the entire line.

### 4.3. Transcript Alignment

As already mentioned, the transcript alignment is intertwined with the word segmentation, so that a word segmentation is performed first, but the bounding boxes it provides are either validated or modified, depending on whether their sizes are deemed as consistent with the number of characters of the hypothesised transcript.

**Figure 7:**
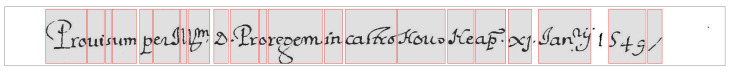
Examples of preliminary word segmentation: the bounding boxes are represented as grey areas of the text line with red contours.

The word segmentation is achieved by computing the Vertical Projection Profile (VPP) of the black pixels of the text line, and each bounding box’s margins are in correspondence with the white pixels’ columns. In this way, different bounding boxes are identified for each of the continuous ink components on the line, as shown in Figure 7.

The word segmentation provides the sequence of bounding boxes *W*:

$$W=<w_{1},w_{2},\ldots ,w_{m}>$$

where *m* is the number of identified boxes. For each text image, its digital transcription is available, from which it is possible to construct the sequence of transcripts *T*:

$$T=<t_{1},t_{2},\ldots ,t_{n}>$$

where *n* is the total number of words that make up the transcription of the line of text. Aligning each bounding box in *W* with its corresponding transcript in *T* would be a trivial task in case m=n and each bounding box includes just a one-word image, i.e., if an error-free word segmentation would be available. As such an ideal word segmentation is not available, there may be both over- and under-segmentation errors. To deal with them, the alignment algorithm involves a correction process that, in short, attempts at either merging adjacent bounding boxes or splitting a bounding box to delineate whole word images. The method consists of scanning the sequences *W* and *T* for analysing each ordered pair ($w_{curr},t_{curr})$ and performing a consistency test to decide whether it is possible to align the transcript with the bounding box.

To perform the consistency test, the algorithm computes the *Average Character Width (ACW)* for each line of text according to the following equation:

$$ACW=\;\frac{\sum_{W}(Word\;image\;width\;(pixels))\;}{\sum_{T}(Number\;of\;characters)\;}$$

It also records the minimum and maximum value of ACW calculated over all the text lines extracted from the document respectively denoted in the following with mth and Mth. Then, assuming that the transcript to be linked with $w_{i}$ is $t_{j}$, it estimates the ACW for the box $w_{i}$ as:

$${ACW}_{box}=\;\frac{width\;of\;w_{i}}{number\;ofcharacter\;of\;t_{j}}$$

The value of ${ACW}_{box}$ is then compared with the values of mth and Mth, so to distinguish three different cases:

1. **Correct segmentation: $mth<{ACW}_{box}<Mth$.** The size of the box $w_{i}$ matches the number of characters in the transcript $t_{j}$. In this case, the transcript $t_{j}$ is aligned with the bounding box $w_{i}$, and the next unmatched pair $\left(w_{i+1},t_{j+1}\right)$ is considered.
2. **Over-segmentation: ${ACW}_{box}<mth$.** The box size $w_{i}$ is too small to accommodate the number of characters in the transcription $t_{i}$. In this case, the algorithm assumes that an over-segmentation error has occurred, and the tentative word segmentation is modified by merging $w_{i}$ with $w_{i+1}$. This way, the box size increases, and the consistency test can be repeated. If it is passed, the merged bounding boxes are associated with the transcript $t_{j}$, and the next unmatched pair $\left(w_{i+2},t_{j+1}\right)$ is considered.
3. **Under-segmentation: ${ACW}_{box}>Mth$.**. The box size $w_{i}$ is too large to accommodate the number of characters of the transcript $t_{j}$. In this case, the algorithm assumes that an under-segmentation error has occurred.

In our previous work [33], we dealt with this case by merging $t_{j}$ with the adjacent transcription $t_{j+1}$, computing the ${ACW}_{box}$ using the total number of characters of $t_{j}$ and $t_{j+1}$ and then performing the consistency test. If successfully passed, the bounding box $w_{i}$ was associated with the two transcripts $t_{j}$ and $t_{j+1}$.

In this work, before attempting the merging of the transcripts, we have added a step for attempting to split the bounding box. For this purpose, the box $w_{i}$ is tentatively split in correspondence to the minimum of its VPP so that it is possible to segment within the ink trace. The leftmost part of $w_{i}$ and the transcript $t_{j}$ are then considered for the consistency test. If it is passed, the split and the corresponding alignment are validated, the transcripts merging is skipped, and the remaining part of the box $w_{i}$ and the transcript $t_{j+1}$ are considered for the next consistency test. Otherwise, the transcripts’ merging is performed.

**Figure 8:**
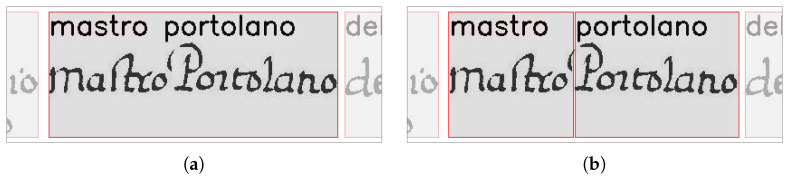
Example of correction of under-segmentation errors: (**a**) the results of the original algorithm show that the alignment assigned a single box to the transcription of the two words “mastro Portolano”; (**b**) the results of the modified algorithm show that it was able to split the bounding boxes and assign them to the two transcripts.

Figure 8 shows an example of the results produced by the change we have introduced to deal with under-segmentation errors. It shows that the modified algorithm is able to correct the word under-segmentation error and eventually correctly align the corresponding transcriptions.

The alignment, thus, proceeds by going through the sequences *W* and *T* and performing a consistency test between a potential box and a transcript from time to time. The original method provides two options for the order in which pairs are to be selected for consistency testing. The first method is called Forward and consists of selecting the boxes and the transcripts from left to right in the sequences. The second method is called MiM (Meet in the Middle), where we alternately select the text line sides. In short, we start with the leftmost box/transcript and perform the consistency test. Once this is performed, instead of continuing with the next box/transcript, we go to the rightmost box/transcript and move one step backwards, from right to left. We then return to the leftmost box/transcript among those still waiting to be aligned and so on. In this way, we try to limit the spread of alignment errors to the entire line of text and avoid a possible “snowball effect”.

**Figure 9:**
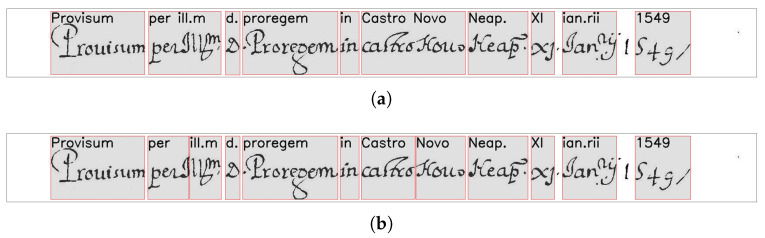
Examples of alignment: (**a**) the result of the original MiM algorithm; (**b**) the result of our alignment method.

Figure 9 shows an example of the entire process of alignment. Comparing Figure 9a,b, where the first is the result of the alignment obtained with the original MiM method, and the latter is the result carried out with our method, we can observe that the original method provided a single box for the two transcripts *“Castro Novo”*, while the new method returns a box for each transcript.

## 5. Results

To assess the performance of the proposed solution, we have used the colour images corresponding to the recto/verso of five paper documents of the *Moccia Code*, referred to as *ff. 6r–10r*, whose line-by-line transcription is available in [21], and processed them according to the workflow depicted in Figure 2. To assess the contribution to the performance of each of the main components of our solution, in the following, we report first the performance of the line segmentation and then the performance of the transcript alignment modules.

### 5.1. Line Segmentation

Table 1 reports the segmentation results on the Moccia Code achieved by our method, and by the method, in [32], which serves as an inspiration for our work. For a fair comparison with the state of the art, the table also includes the performance of the methods proposed in [34,35,36], although they adopt deep-learning techniques. The first one has been chosen because its performance compares favourably with the top methods proposed at the two most recent competitions on text-line segmentation, and therefore it is assumed to represent the state of the art. The other two because the trained (on a large amount of data) models they produce can be used “off-the-shelf”, with no further training, thus fitting the application scenario we are dealing with. Moreover, as the implementation of the first method is also publicly available, we have reported the performance achieved by our method and its competitors on the Bentham [37], the George Washington [38] and the Jefferson Letter [9] datasets, as they fall within the same historical period as the Moccia Code, and the Saint Gall [39], although it contains documents produced in the Middle Age, because it is among the most widely used for performance assessment. It is worth noting that all these datasets include the test set, so that the performance of methods are directly comparable.

### 5.2. Alignment Method

The performance of an alignment method are usually reported in terms of accuracy, i.e., the ratio between the number of times the handwriting in the word image corresponds to its transcript and the total number of transcripts to align, as mentioned in Section 2. For implementing such a definition, the basic idea is that of evaluating, for each transcript, the overlap between the word image in the ground truth and the one produced by the system, and assuming that the word images correspond to its transcript whenever the overlap with the ground truth image is above a threshold. This way of evaluating the accuracy, however, is blind to the actual sizes and positions of the bounding boxes. On the contrary, and depending on the application, it does matter whether or not the non-overlapping area contains a relevant part of the ink, so that the reported accuracy may not reflect the actual accuracy.

**Figure 10:**
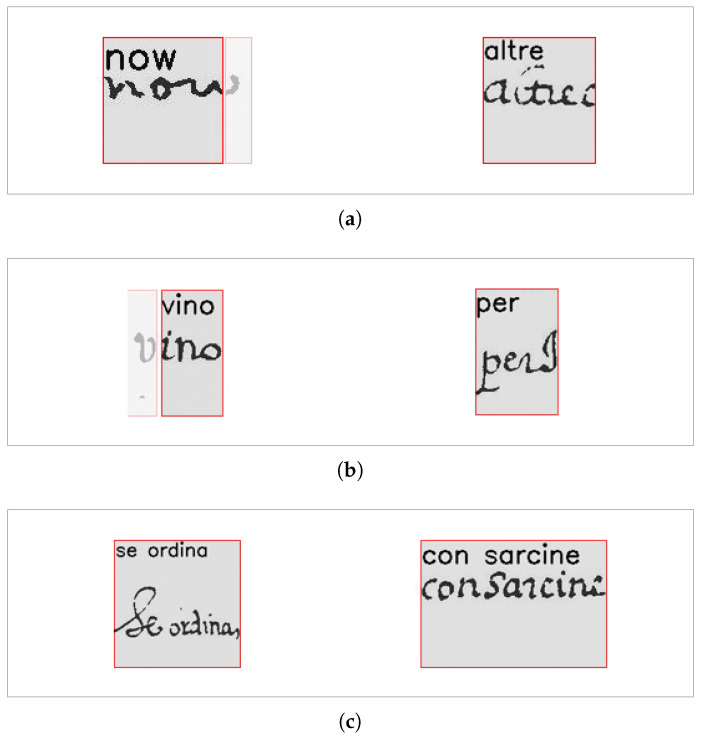
Examples of acceptable alignments: (**a**) missing/added strokes; (**b**) missing/added characters; (**c**) multiple-to-one alignment.

To overcome this drawback, we have implemented the performance assessment by visual inspection, assuming that a transcript has been correctly aligned with a bounding box if the word image within the bounding box contains the ink corresponding to the writing of *all* the characters of the transcript. This condition will be referred to in the sequel as the *perfect* alignment. This is a very restrictive condition, as it leads to consider as wrong alignment the cases shown in Figure 10a, even though the missing/added part of the ink does not prevent one to read the transcript. Thus, we have considered a less restrictive one, referred to hereinafter as *acceptable* alignment, assuming that a transcript is correctly aligned even when the bounding box misses *at most* the ink of the starting/ending character of the transcript, or when it contains, in addition to the ink corresponding to the transcripts, the ink of at most the starting/ending character of the adjacent transcripts. Such condition allows for counting as correct alignment the case depicted in Figure 10b, where the bounding boxes miss or include the ink corresponding to one character.

Eventually, it is worth noting that this condition allows two or more transcripts to be counted as aligned even if they are aligned within the same bounding box. Figure 10c shows examples where under-segmentation errors cannot be corrected. In these cases, the alignment algorithm merges the transcripts, assuming that the ink of both of them is within the bounding box (which is correct in both cases), although it is not possible to split it reliably in non-overlapping regions (which is the case depicted at its best in the right image in Figure 10c) Thus, the solution is acceptable according to our definition because the bounding box includes all the ink of each word.

To mitigate as much as possible the bias introduced by the subjective judgement intrinsic to our definition, the evaluation was carried out independently by two subjects. After the evaluation, whenever the evaluations of the same alignment by the experts were different, the final decision was left to a third subject.

Table 2 reports the performance achieved on the Moccia Code when either perfect or acceptable alignment is adopted. In order to compare our method with the state of the art, in Table 3 we list the alignment methods proposed in the literature in the past twenty years. For each method, we report the dataset they used, the type of writing of the documents, the historical period of production, whether or not it is available, either publicly on by request to the collectors, the method used for text alignment, and the reported performance. As the table shows, some datasets are not available, and others contain the digital images of the documents, but the transcription of the content is not available (and we were not able to provide it ourselves because of the language). Two of them contain early printed or ciphered documents, while the Saint Graal contains documents produced in the 9th century, which is eight centuries earlier than the Moccia Code. Moreover, there are no available implementations of the methods, and the related papers do not provide all the details for a re-implementation. Eventually, in the case of the Corpus Cristo Salvador, the composition of the test set used during the performance assessment is not reported in the paper, nor it is identified in the data set. All these issues restrict the possibility of a direct comparison to the methods that have been tested on the Bentham, the George Washington, and the Jefferson Letter data sets because the test sets are publicly available in the repositories. Table 4 reports the results of our method and those of its competitors. Let us remark that, for a fair comparison with the metrics used in the other studies, we report the results of our method in terms of perfect alignment accuracy.

## 6. Discussion

**Figure 11:**
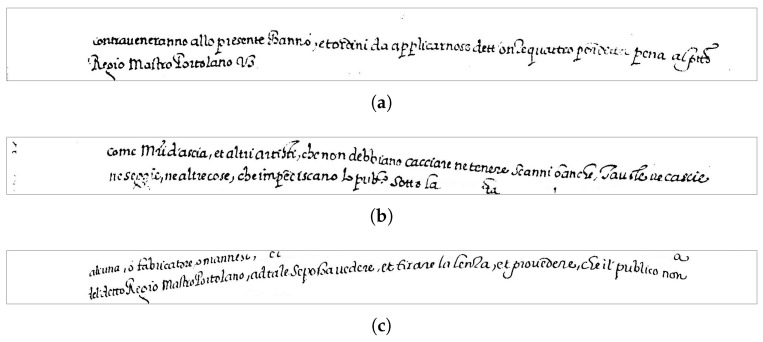
Line segmentation improvements. A few examples of line segmentation errors introduced by the competing methods that are correctly segmented by our method. (**a**) The entire ink of the next line is included in the current line, (**b**) some of the ink from the next line is included in the current line, (**c**) some of the ink from the previous line is included in the current line.

The results of the line segmentation reported in Table 1 show that the proposed method outperforms both the learning-free method proposed in [32] and the learning-based one recently proposed in [34], albeit to a different extent, and that in both cases the largest improvements are achieved on the Moccia Code. This is due to the text lines with a curved baseline of this collection, which none of the two methods can deal effectively, as they tend to merge several lines whenever the baseline of one of them bends along the writing direction, as it happens on the page image shown in Figure 11.

**Figure 12:**
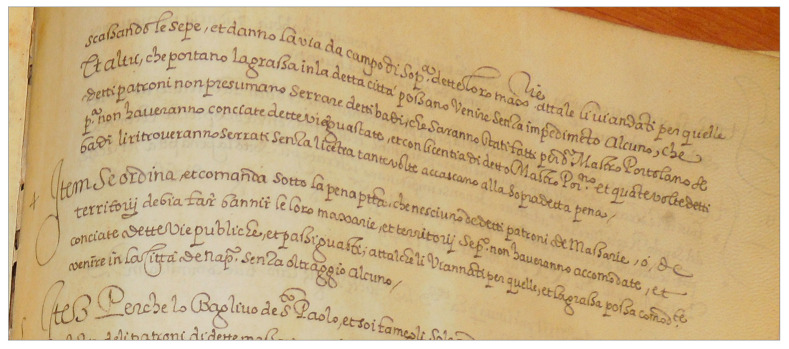
Line segmentation errors An example of a page whose lines of text baseline exhibit a non-uniform and very pronounced bending, leading all the methods, including the one here proposed, to fail.

They also show that the docExtractor and the dhSegment methods exhibit the top performance, as they are effective even on the page shown in Figure 12, where all the other three methods, including the one presented in this paper, fail. However, it is noteworthy that docExtractor provides as result the region including only the centre zone of the handwriting, thus ignoring both ascenders and descenders, while dhSegment outputs only the baseline. In the case of applications requiring all the ink of the text-line to be made available to the following steps, as in our case, some ad-hoc post-processing must be developed and integrated within the workflow of the application, so that their adoption may not represent the best option.

The results reported above have been achieved by dividing the text area into eight stripes, and this value has been empirically chosen by observing on the document of the Moccia Code that it corresponds to stripes whose ink contains at least one word for each text line, so as to make the baseline estimation more reliable. The results achieved on other datasets confirm this can be assumed as a fixed value for documents produced in the same historical period, and that it may also work for older documents.

As regards to the alignment, Table 2 shows that there is not a clear indication of which alignment strategy performs better between the Forward and the Mim variants we have implemented. The former outperforms the latter in terms of perfect alignment, while the opposite is true, although to a limited extent, in terms of acceptable alignment.

**Figure 13:**
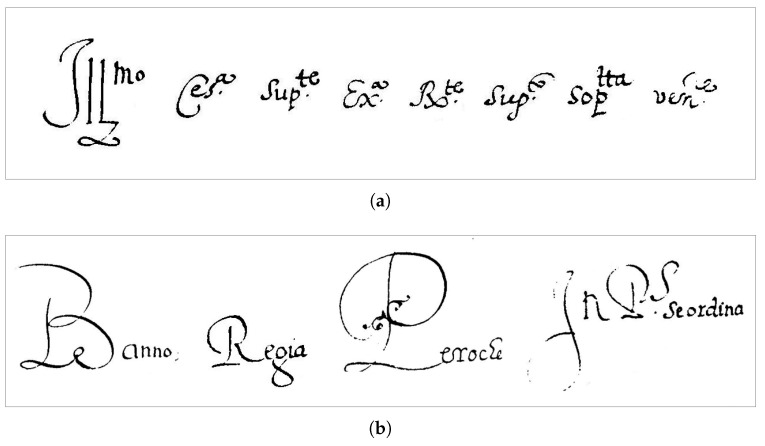
Some examples of abbreviations and uppercase letters in the Moccia Code dataset: (**a**) abbreviations; (**b**) flourishing uppercase letters.

The error analysis has shown that most of the alignment errors are due to irregularity in both inter-word spacing and the size of the bounding boxes that contain the word image of the same transcript. As a matter of fact, we have observed that most of the errors are located along text lines that, as the writing approaches the rightmost margin of the page, show a shrinking/expansion of either the size of the inter-word gaps or the horizontal extension of the writing, very likely to allow writing an entire word before the margin. There are also many abbreviations in the text where the last part of the word is displayed in superscript (Figure 13a). This affects the pixel size of the image of the abbreviated word and makes the alignment more complex for the method.

Another feature of the collection is that the first or the last letter of a paragraph are often in uppercase and/or flourished (Figure 13b). This change in writing style also complicates automatic alignment by the method. Whenever a line of text shows these features, the method fails to align the leftmost and/or the rightmost transcript of the line, and the errors propagate from either sides of the line. However, the better results achieved by both methods in terms of acceptable alignments, show that word-segmentation errors are mostly due to bounding boxes missing (or containing) at most the ink of one character (belonging to the adjacent transcripts) and that the MiM strategy is slightly better than the forward one in dealing with segmentation errors happening simultaneously at both sides of the text line.

## 7. Conclusions

We have presented a fully automatic solution for aligning the transcription of small collections of historical handwritten documents with the digital images of the documents. To fulfil these requirements, we have presented a learning-free, end-to-end approach that incorporates a line segmentation algorithm and an alignment algorithm intertwined with a word segmentation step. The former can extract text lines with curved baseline, the latter can detect and correct both over- and under-segmentation errors introduced by the word segmentation step.

The performance of the proposed solution has been evaluated on the Moccia code, a small collection of historical handwritten documents that proved to be a very challenging testbed in comparison with some of the datasets currently used as benchmarks for performance assessment.

The performance of the line segmentation algorithm shows that it outperforms the state of the art, with the only exception of the docExtractor and the dhSegment, but none of them provides as output all the ink of the handwriting of the text line, thus limiting the possibility of using them without further ad-hoc provisions as in our case, as well as for keyword spotting, writer/script identification and handwriting recognition.

The results also suggest a possible improvement to make the algorithm more effective and less computationally demanding. As mentioned in Section 4.2, the algorithm works by dividing the text area into eight stripes. Although this setting, empirically chosen from observation on the Moccia Code, has been proved to be reasonable also for the other data sets on which we have evaluated the performance, it may not work effectively on other collections presenting different writing styles and using different languages. To overcome this drawback, we are currently working on a self-adaptive algorithm that starts by setting S=1, and in case the number of detected lines is lower than expected, increments progressively *S* until the segmentation leads to the expected number of lines, or when the values of *S* is such that the width of the stripes is smaller than the average size of a character, which is considered as the smallest piece of ink to whom a baseline can be associated.

As with regards to the alignment performance, the results of a direct comparison with the learning-free state-of-the-art methods [9,10,11,33] have shown that the proposed method performs better than its competitors on every dataset on which they have been tested. They also show that, at least qualitatively, they are in line with the performance reported by other state-of-the-art methods on datasets not available for a direct comparison. Eventually, it is worth noting that in the case of the Moccia Code, the performance of our method is lower than those achieved on the datasets currently used in the literature as a benchmark for performance assessment, confirming that the Moccia Code represents a much more challenging testbed for text alignment of mostly textual handwritten documents.

The experimental results achieved so far not only showed that our methods represent an effective fully automatic solution for text alignment of mostly textual historical handwritten documents, but they also suggested one more possible future development. When the results of the consistency test indicate that an under- or an over-segmentation error has occurred, the alignment algorithm attempts to correct it by exploiting the information carried by the adjacent bounding box/transcript. Even when, at the end of the process, it becomes evident that something went wrong because either a transcript or a bounding box is left unaligned, the algorithm does not allow backtracking the previous decisions for attempting alternative word segmentation. Thus, our next step in this direction would be that of designing and implementing such a backtracking mechanism, taking into account that the number of bounding boxes to align should be the same as the number of words of the line transcription. Eventually, the consistency check itself can be made more effective by estimating the average character width for each character class and then using such value to estimate the width of the box to be used in the consistency check.

## Figures and Tables

**Table 1:** Line segmentation results. The table reports, for each data set, the actual number and the number of correctly segmented text lines provided by each method.

Dataset	N Lines	Our	Surinta et al. [32]	Alberti et al. [34]	docExtractor [35]	dhSegment [36]
Moccia	275	253	153	144	274	267
Code		92.00%	55.64%	52.36%	99.64%	97.09%
Bentham	1056	1004	924	1003	1040	967
Collection		95.08%	87.50%	94.98%	98.48%	92.45%
George	653	600	585	587	632	635
Washington		91.88%	89.59%	89.89%	96.78%	97.24%
Jefferson	23	22	19	19	22	23
Letter		95.65%	82.61%	82.61%	95.65%	100.00%
Saint Gall	1430	1415	1351	1387	1419	1420
		98.95%	94.48%	96.99%	99.23%	99.30%

**Table 2:** Performance of the alignment method and its variants on the Moccia Code. The results are given in terms of accuracy. The best results are in boldface.

Alignment	Forward	MiM
*Perfect*	**45.05 %**	42.47%
*Acceptable*	67.59 %	**68.39%**

**Table 3:** Overview of alignment methods in the literature. The period of production is expressed by the ordinal of the century AC. Results are reported in terms of accuracy.

	Type	Dataset	Period	Available	Method	Result
[9]	Handwritten	Thomas Jefferson Letter	XVIII	Yes	Dynamic Programming	72.00%
[10]	Handwritten	George Washington	XVIII	Yes	Dynamic Time Warping	75.40%
[11]	Handwritten	George Washington	XVIII	Yes	HMM	72.80%
[12]	Handwritten	Corpus Cristo Salvador	XIX	Yes	HMM	92.80%
[14]	Handwritten	The Swiss Literary Archives	XX	No	HMM	94.66%
[13]	Handwritten	Kabinet van de Koningin (KdK) collection	XIX	Only images Transcription not available	Ink Projection Segmentation	69.00%
[15]	Handwritten	ICDAR20009 test set	XXI	No	Word Segmentation	97.04%
[16]	Handwritten	ICDAR20009 test set	XXI	No	Word Segmentation	99.48%
[17]	Handwritten	Queste del Saint Graal	IX	Yes	Segmentation Free	72.90%
[18]	Handwritten	C5 Hattem Manuscript	XVI	Only images Transcription not available	HMM—Dynamic programming	75.50%
[33]	Handwritten	Bentham Collection	XVIII	Yes	Ink Projection	75.93%
[19]	Early Printed	Gutenberg Bible	XV	Yes	CNN-based	90.00%
[20]	Chipered	Copiale ciphered manuscript	XVIII	Yes	Attention	90.00%

**Table 4:** Comparison of alignment results with the state of art. The results are given in terms of accuracy. The best results are in boldface.

Database	Method	Result
Bentham Collection	[33]	75.93%
	our	**77.20%**
George Washington	[10]	75.40%
	[11]	72.80%
	our	**79.76%**
Jefferson Letter	[9]	72.00%
	our	**88.80%**

## Data Availability

Not applicable.
